# Stoichiometry-Defined Phase Selection and Hydrophobic Molecular Sealing in Bioderived Deep Eutectic Solvents

**DOI:** 10.1021/acsmaterialsau.6c00088

**Published:** 2026-06-25

**Authors:** Shunsuke Yamada, Takashi Honda

**Affiliations:** † Department of Electrical and Electronic Engineering, 12924 Kyushu Institute of Technology, 1-1 Sensuicho, Tobata, Kitakyushu, Fukuoka 804-8550, Japan; ‡ Center for Advanced Organic-Inorganic Hybrid Materials, Kyushu Institute of Technology, 1-1 Sensuicho, Tobata, Kitakyushu, Fukuoka 804-8550, Japan

**Keywords:** bioderived deep eutectic solvents, stoichiometric control, phase behavior, hydrogen-bond network, moisture resistance

## Abstract

Deep eutectic solvents (DESs) are promising ionic media owing to their abundance, ionic conductivity, and wide potential windows. Bioderived DESs based on choline chloride are attractive due to their biodegradability and biocompatibility; however, their strong hygroscopicity leads to severe moisture uptake and instability. Here, we report a bioderived moisture-resistant DES of acetylcholine chloride as a hydrogen bond acceptor (HBA) and 3-phenylpropionic acid as a hydrophobic hydrogen bond donor. The hydrophobic component acts as a molecular seal, shielding the hygroscopic HBA from moisture through hydrogen-bond network reconfiguration. Systematic variation of the molar ratio reveals four distinct regimes: immiscible mixtures (1:1), saturated states (1:1.5), a homogeneous glass-forming liquid at the eutectic composition (1:2, *T*_g_ = −57.9 °C), and crystallizable phases at higher HBD contents (1:3 and 1:4). Only the eutectic composition stabilizes a uniform liquid without observable melting transitions, exhibiting over 75% reduction in moisture uptake relative to the parent salt at 58% relative humidity. The DES demonstrated a high ionic conductivity (197 μS cm^–1^) and a wide potential window (2.43 V). Moreover, the DES exhibited high biodegradability (>90%), exceeding the OECD criterion for ready biodegradability (>60% within 28 days). These findings establish stoichiometry as a governing parameter coupling phase stability with moisture affinity in robust, biodegradable DESs.

## Graphical abstract

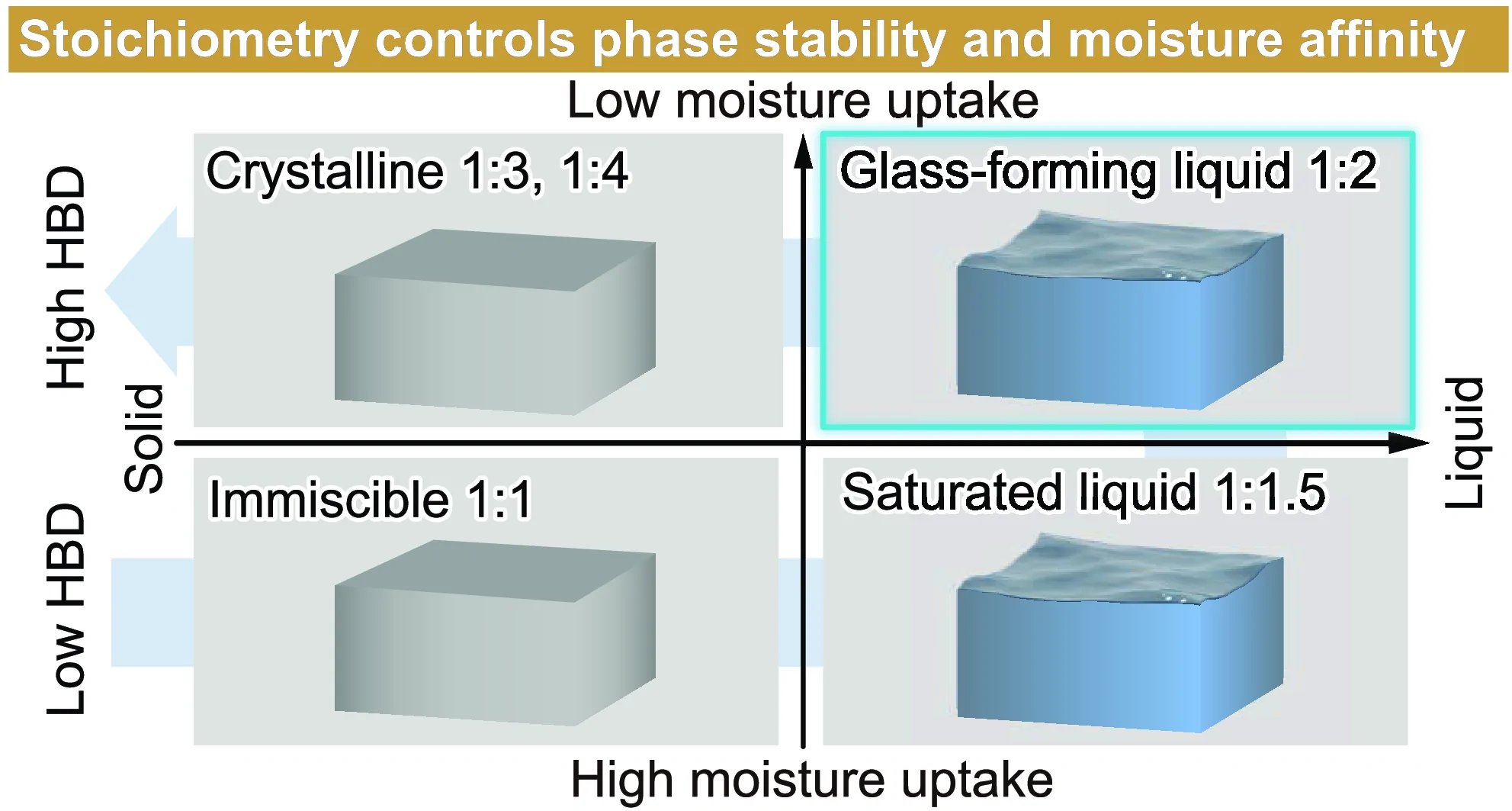

## 1 Introduction

Sustainable ionic materials are increasingly sought for energy storage, electrochemical systems, and green technologies.^1–6^ Deep eutectic solvents (DESs) have emerged as versatile green alternatives to conventional ionic liquids owing to their low cost, ease of synthesis, and tunable physicochemical properties.^7^ In particular, the biocompatibility and potential biodegradability of choline-based DESs, which are derived from naturally abundant components, have attracted increasing research attention.^8,9^ However, choline-based systems are often highly hygroscopic due to strong interactions between choline salts and water, resulting in uncontrolled water uptake under ambient conditions.^10,11^ In choline-based DESs, the choline salt serves as a hydrogen bond acceptor (HBA) while the coformer acts as a hydrogen bond donor (HBD); both components exhibit affinity for water, with the HBA being particularly susceptible to moisture absorption due to its ionic nature. This behavior compromises phase stability, rheological properties, ionic transport, and reproducibility. Although hydrophobic components have been incorporated to mitigate the moisture sensitivity of such systems, this approach often relies on empirical optimization rather than systematic compositional design.^12–14^ Moreover, limited attention has been paid to quantitatively understanding moisture uptake, its compositional dependence, and its effect on environmental compatibility.^15–19^ More fundamentally, DES research has largely emphasized eutectic melting and liquid formation. In hydrogen-bonded ionic systems, stoichiometry governs more than melting point depression; it dictates phase selection, intermolecular network organization, and emergent physicochemical properties. Nevertheless, how stoichiometry governs transitions among immiscible, glass-forming, and crystalline states in DESs—and how these states correlate with moisture affinity—remains unclear. Consequently, rational design principles that couple phase stability with environmental robustness for bioderived DESs are lacking. We hypothesize that a hydrophobic HBD can act as a molecular seal, simultaneously governing phase selection and suppressing moisture uptake.

Here, we report a bioderived moisture-resistant DES (BMDES) composed of acetylcholine chloride ([Ach]­[Cl]) as an HBA and 3-phenylpropionic acid ([Ph]) as an HBD (Figure 1a), selected for their low toxicity,^20^ renewable origin,^21,22^ and Generally Recognized As Safe status of [Ph].^23^ The hydrophobic HBD acts as a molecular seal shielding the hygroscopic HBA from moisture through hydrogen-bond network reconfiguration. Systematic molar ratio variations revealed four distinct regimes: an immiscible mixture (1:1), a saturated state (1:1.5), a homogeneous glass-forming liquid at the eutectic composition (1:2), and crystallizable phases at higher HBD contents (1:3 and 1:4). Remarkably, only the 1:2 composition stabilized as a uniform liquid without observable melting transition events within the measured temperature range (Figure 1b) and exhibited suppressed moisture uptake under ambient conditions (Figure 1c). Spectroscopic and structural analyses indicated that stoichiometric balance reconfigured the hydrogen-bond network and altered ion–molecule interactions, thereby coupling phase selection with moisture affinity. These findings establish stoichiometry as a governing parameter for discrete phase stabilization in bioderived DESs, providing a foundation for the rational development of moisture-stable ionic materials for wearable electronics,^24–27^ environmental monitoring,^27–29^ and energy storage systems.^30–32^ This design principle may be extended to other bioderived ionic systems, enabling the systematic tuning of phase stability and environmental compatibility for sustainable electrochemical technologies, including flexible sensing platforms.

**Figure 1:**
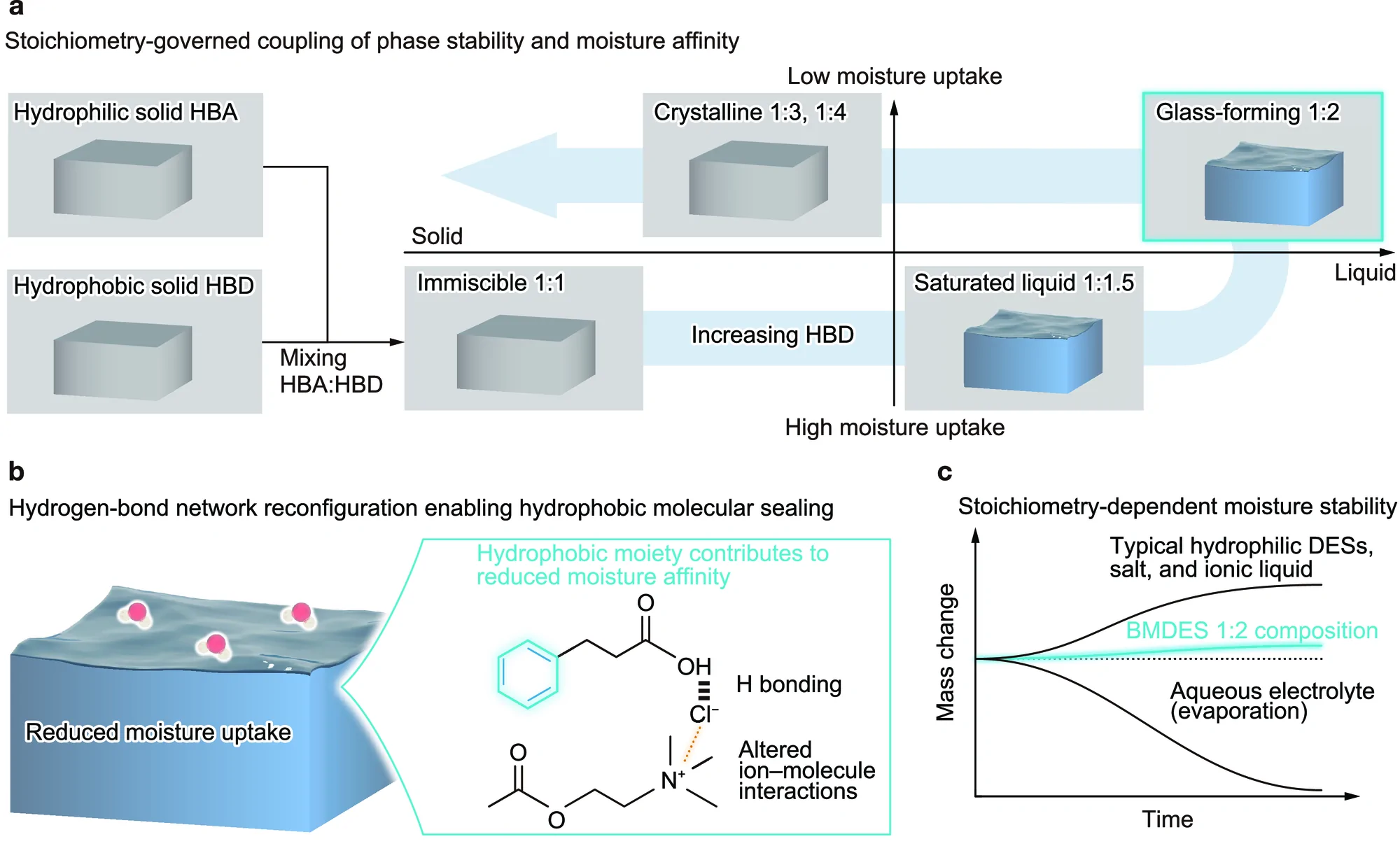
Conceptual framework of stoichiometry-governed phase stabilization and moisture modulation in BMDESs. (a) Schematic illustration of stoichiometric coupling between phase regime and moisture affinity as a function of the molar ratio of HBA to HBD. (b) Proposed hydrogen-bond network reconfiguration at the eutectic composition, highlighting altered ion–molecule interactions and reduced moisture affinity arising from short-range structural organization. (c) Representative schematic of stoichiometry-dependent moisture stability, comparing moisture uptake profiles for hydrophilic ionic materials, an aqueous electrolyte (evaporation-dominated), and the eutectic composition (1:2).

## 2 Results and Discussion

### 2.1 Stoichiometry-Dependent Phase Behavior of Acetylcholine-Based DES Systems

The BMDES systems were prepared in glass vials at molar ratios of 1:1, 1:1.5, 1:2, 1:3, and 1:4 ([Ach]­[Cl]:[Ph]), denoted as BMDES1, BMDES1.5, BMDES2, BMDES3, and BMDES4, respectively, and annealed at 80 °C until the solids turned into liquids (Figure 2a,b). BMDES1 was visibly heterogeneous, appearing as an opaque suspension even after prolonged heating, indicating incomplete miscibility between [Ach]­[Cl] and [Ph] at equimolar composition. BMDES1.5 exhibited improved mixing compared to BMDES1 (Figure S1a and S1b), forming a viscous, homogeneous liquid. However, slight recrystallization of [Ach]­[Cl] was observed upon cooling to room temperature (∼25 °C), suggesting a saturated compositional regime. By contrast, BMDES2 formed a transparent and homogeneous liquid, which remained stable at room temperature without visible phase separation. Notably, BMDES2 remained a transparent liquid even after storage at −20 °C and did not ignite upon exposure to direct flame, indicating notable thermal stability (Figure S2a and S2b). BMDES3 and BMDES4 were transparent liquids at elevated temperature; however, crystallization occurred upon cooling or contact with solid surfaces or foreign particles (Figure S3), suggesting enhanced susceptibility to heterogeneous nucleation at higher HBD content. Differential scanning calorimetry (DSC) was performed to investigate the thermal nature of these systems. Samples were cooled to −80 °C and subsequently heated to 100 °C at a heating rate of 10 °C min^–1^ (Figure 2c). DSC measurement of pure [Ach]­[Cl] was not performed due to its strong hygroscopicity, which precludes reliable thermal characterization under ambient conditions. Pure [Ph] exhibited a sharp melting endotherm at 51.1 °C, consistent with its crystalline nature. The supernatant obtained from BMDES1.5 after centrifugation exhibited a glass transition at −52.6 °C, indicating the presence of a glass-forming liquid fraction within the otherwise heterogeneous system. BMDES2 displayed a glass transition at −57.9 °C, without detectable crystallization or melting events in the measured temperature range (−70 to 95 °C), indicating a homogeneous glass-forming liquid. BMDES3 exhibited a glass transition at −59.2 °C, followed by an exothermic crystallization peak at −5.11 °C and an endothermic melting peak at 26.7 °C upon reheating. Similarly, BMDES4 exhibited a glass transition at −61.5 °C, an exothermic crystallization peak at −24.6 °C, and a melting endotherm at 38.7 °C. The absence of crystallization and melting transitions for BMDES2, in contrast to the well-defined thermal events observed for BMDES3 and BMDES4, indicated the discrete stabilization of a glass-forming liquid at the 1:2 stoichiometry. The phase behavior of these samples is summarized in Figure 2d based on the crystallization temperature (*T*_c_), melting temperature (*T*_m_), and glass transition temperature (*T*_g_) determined from DSC measurements. The resulting thermal transitions revealed distinct phase regimes induced by stoichiometric variations, including glassy, supercooled liquid, and crystalline states. BMDES1 corresponded to an immiscible regime, in which incomplete mixing persisted even at elevated temperatures; BMDES1.5 represented a saturated state with partial homogenization but residual heterogeneity; BMDES2 uniquely stabilized as a homogeneous glass-forming liquid without observable melting transitions, indicating eutectic behavior within the investigated composition range. Among the investigated compositions, BMDES2 exhibited a distinct glass transition temperature (*T*_g_ = −57.9 °C) and was the only composition that remained as a homogeneous glass-forming liquid without crystallization or melting events; and BMDES3 and BMDES4 corresponded to the crystallizable regime, undergoing a glass transition at low temperature followed by cold crystallization and subsequent melting upon reheating.

**Figure 2:**
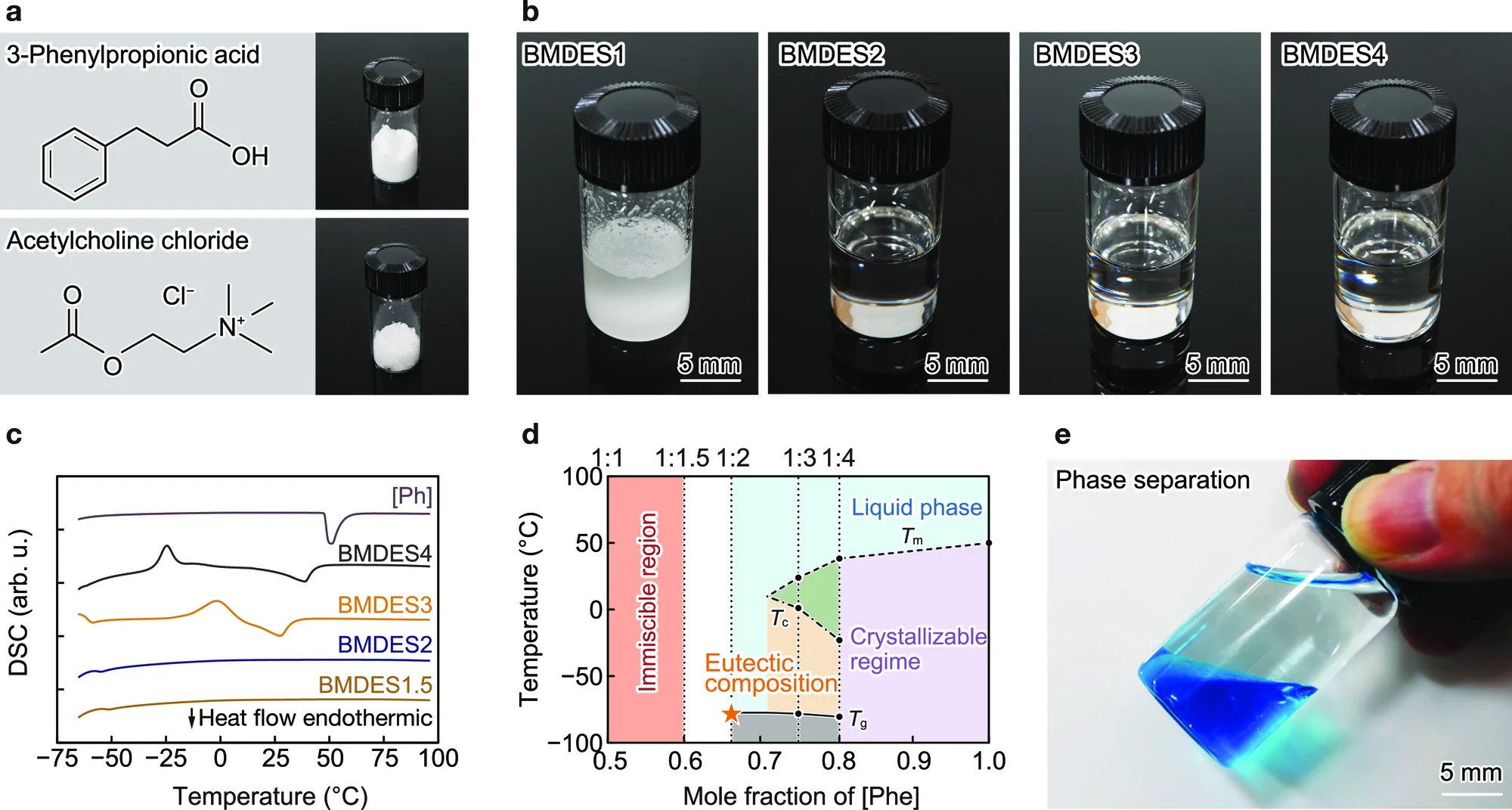
Stoichiometry-dependent phase behavior of BMDES systems. (a) Chemical structures and photographs of acetylcholine chloride ([Ach]­[Cl]) and 3-phenylpropionic acid ([Ph]). (b) Photographs of BMDES samples at different molar ratios after annealing at 80 °C. (c) DSC thermograms illustrating distinct thermal signatures for different BMDES compositions, highlighting the exclusive glass-forming behavior at the 1:2 eutectic composition. (d) Compositional phase map summarizing immiscible, saturated, glass-forming, and crystallizable regimes identified from visual and thermal analyses. (e) Photograph illustrating stoichiometry-dependent phase partitioning upon contact with DIW. BMDES2 was dyed blue for visual clarity.

To evaluate the compositional dependence of water interaction, BMDES1.5, BMDES2, BMDES3, and BMDES4 were introduced into deionized water (DIW) at room temperature. BMDES1 was excluded due to phase heterogeneity, and the supernatant of BMDES1.5 was employed after centrifugation. Upon mixing with DIW, biphasic systems formed in which a white emulsion was observed in the upper layer while the BMDES-rich phase remained in the lower layer (Figure S4a). The emulsion layer gradually settled and coalesced into a clear aqueous phase within 24 h (Figure S4b). BMDES2 formed a distinct secondary phase without observable bulk dissolution under static conditions, yielding a stable biphasic structure (Figure 2e). Results indicated that homogeneously mixed BMDES compositions exhibited hydrophobic behavior, resisting bulk dissolution in water and forming stable biphasic systems, thereby motivating a systematic investigation of hygroscopic behavior under controlled humidity conditions.

### 2.2 Hydrogen-Bond Network Reconfiguration and Structural Analysis

Spectroscopic and structural analyses were performed to elucidate the molecular origin of the stoichiometry-dependent phase behavior. Fourier-transform infrared (FTIR) spectroscopy revealed variations across the measured range of 500–3500 cm^–1^, indicating composition-induced changes in the hydrogen-bonding environment (Figure 3a). The carbonyl stretching region provided direct insight into reconfiguration of the hydrogen-bond network (Figure 3b). The FTIR spectrum of pure [Ph] displayed a sharp C=O band at 1693 cm^–1^, characteristic of the carboxylic acid group, whereas that of pure [Ach]­[Cl] displayed an ester carbonyl band at 1731 cm^–1^. In the FTIR spectra of BMDES2 and BMDES3, these distinct absorptions were replaced by a single broad band centered at approximately 1725 cm^–1^. The disappearance of individual carbonyl peaks and emergence of a single broad band indicated modification of the carbonyl environment, consistent with hydrogen-bond formation. Additional spectral changes in the fingerprint region (900–1500 cm^–1^) supported reorganization of the hydrogen-bond network. Alterations in the 1370–1430 cm^–1^ region, corresponding to the C–O–H bending modes of [Ach]­[Cl], indicated reconfiguration of the carboxyl environment upon mixing (Figure 3c). Moreover, the emergence of a new band at 1154 cm^–1^ in the FTIR spectra of the BMDES samples, which was absent in the FTIR spectra of the pure components (Figure 3d), was assigned to C–O stretching vibrations associated with a hydrogen-bonding environment. The O–H bending mode at 930 cm^–1^ observed for pure [Ph] disappeared in the FTIR spectra of BMDES2 and BMDES3 (Figure 3e), suggested involvement of the acidic proton in hydrogen bonding. Collectively, these spectral changes—including alterations in C=O stretching and C–O–H bending modes, emergence of a new vibrational feature, and disappearance of free O–H vibrations—supported the formation of a reorganized hydrogen-bond network at the eutectic composition. These findings suggested that stoichiometric balance governed intermolecular organization, enabling discrete phase selection. X-ray diffraction (XRD) was conducted to examine the long-range structural order of pure components and selected BMDES samples (Figure 3f). The XRD pattern of pure [Ph] displayed sharp diffraction peaks, consistent with its crystalline nature. [Ach]­[Cl] was not analyzed owing to its strong hygroscopicity and deliquescent behavior under ambient conditions. The XRD pattern of BMDES1.5 (supernatant after centrifugation) exhibited a broad amorphous halo without Bragg reflections, indicating the removal of crystalline residues and retention of a disordered liquid phase. Similarly, the XRD patterns of BMDES2 and DES3 demonstrated broad halo patterns without distinct diffraction peaks, characteristic of amorphous or liquid-like structures at room temperature. The absence of Bragg reflections was consistent with the disruption of long-range periodicity upon the formation of a hydrogen-bonded ionic network. Combined with the DSC and FTIR results, the XRD results supported stoichiometry-dependent reorganization from crystalline order toward a glass-forming or liquid-like state in eutectic and near-eutectic compositions.

**Figure 3:**
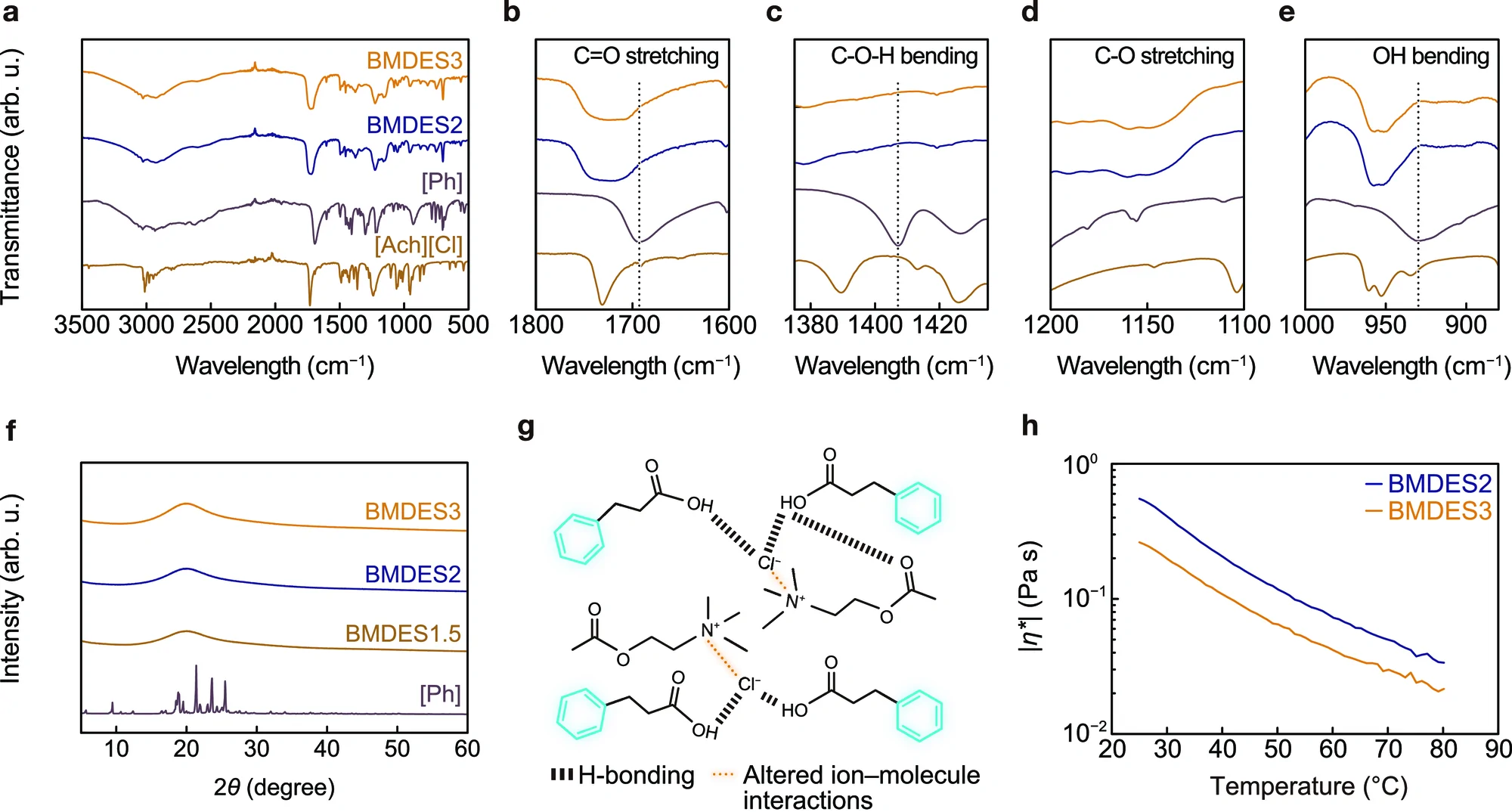
Molecular and structural origin of stoichiometry-dependent phase stabilization. (a) FTIR spectra (500–3500 cm^–1^) of pure components and BMDES samples. (b) Expanded view of the carbonyl stretching region (1600–1800 cm^–1^), showing individual C=O bands and their broadening for BMDES2 and BMDES3. (c) Alterations in the 1370–1430 cm^–1^ region, reflecting modification of the carboxyl environment. (d) Emergence of a new vibrational band in the 1100–1200 cm^–1^ region associated with hydrogen-bond network reorganization. (e) Enlarged O–H bending region (875–1000 cm^–1^), indicating the disappearance of free carboxylic acid vibrations upon BMDES formation. (f) Powder XRD (2*θ* = 5°–60°) patterns showing crystalline peaks for pure [Ph] and broad halo features for BMDES samples. (g) Schematic illustration of the reorganized hydrogen-bond network at the eutectic composition, highlighting short-range ordering and altered ion–molecule interactions. (h) Temperature-dependent viscosity profiles demonstrating compositional modulation of liquid dynamics.

Based on the FTIR and XRD results, a schematic representation of the reorganized intermolecular network at the eutectic composition is proposed in Figure 3g. In this model, chloride anions (Cl^–^) act as central HBA nodes coordinating multiple hydrogen-bond interactions with the carboxylic acid groups of [Ph], while the ionic interactions between Cl^–^ and the acetylcholine cation are partially reorganized relative to the parent salt. Such reconfiguration of ion–molecule interactions is supported by the FTIR spectral changes observed in the carbonyl stretching region and fingerprint region associated with the [Ach]­[Cl] environment. The redistribution of hydrogen bonds and ion–molecule interactions induces short-range organization yet long-range disorganization, consistent with the broad halo observed in the XRD patterns and the absence of crystallization in the DSC thermograms. The reconfigured network stabilizes the glass-forming liquid state in eutectic and near-eutectic compositions.

The rheological properties of BMDES2 and BMDES3 were evaluated to characterize their liquid behavior. As shown in Figure S5a and S5b, the storage modulus (*G*′) of BMDES2 and BMDES3 remained negligible across the examined angular frequency (*ω*) range, with values on the order of 10^–3^–10^–2^ Pa, while the loss modulus (*G*″) increased linearly with rising *ω*. Consequently, the phase angle (*δ* = tan^–1^(*G*″/*G*′)) approached 90°, indicating dominant viscous behavior. The complex viscosity (η*) remained nearly constant over the entire frequency range at 25 °C, with values of approximately 0.54 and 0.27 Pa·s for BMDES2 and BMDES3, respectively. Results confirmed that both systems behaved as Newtonian fluids, consistent with homogeneous liquid-like states (Figures S5c and S5d). The viscosity of BMDES2 and BMDES3 decreased monotonically from 25 to 80 °C (Figure 3h). Notably, BMDES3 maintained lower viscosity than BMDES2 across the temperature range, suggesting that the [Ph] content modulated intermolecular interaction strength within the hydrogen-bond network.

### 2.3 Moisture Stability and Biodegradability

To evaluate the influence of the hydrophobic HBD on moisture uptake, the hygroscopic behavior of the BMDES systems was investigated under controlled temperature and humidity conditions. No discernible morphological changes were observed for pure [Ph], BMDES2, and BMDES3 after exposure to 58% relative humidity (RH) at 25 °C for 48 h (Figure 4a,b). Pure [Ach]­[Cl] underwent deliquescence, transforming from a white crystalline powder into a transparent liquid under these conditions. In BMDES1, residual undissolved [Ach]­[Cl] gradually dissolved upon moisture uptake, leading to a visibly expanded liquid phase. By contrast, BMDES4 crystallized during transfer to the container, consistent with its metastable nature, as illustrated in Figure 2d. Similarly, pure [Ph], BMDES2, and BMDES3 retained their original appearance after exposure to 33% RH, while [Ach]­[Cl] and BMDES1 underwent deliquescence (Figure S6a and S6b). The same compositional dependence was observed after exposure to 75% RH, with [Ach]­[Cl] and BMDES1 undergoing a comparable degree of deliquescence to that observed at 58% RH (Figure S6c and S6d).

**Figure 4:**
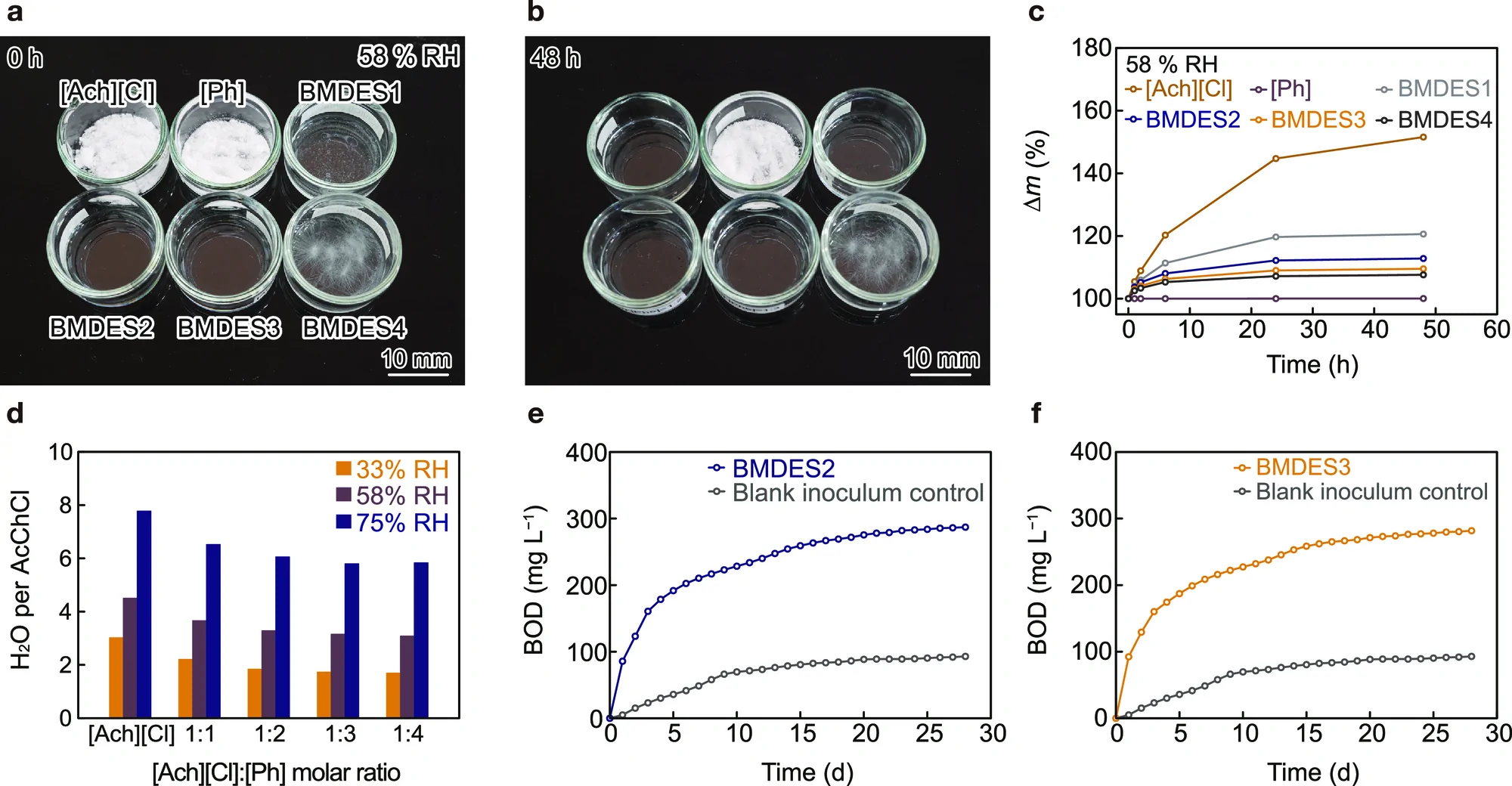
Stoichiometry-dependent moisture stability and biodegradability. Photographs of BMDES samples before (a) and after (b)­exposure for 48 h to 58% RH at 25 °C. (c) Time-dependent mass change of BMDES samples at 58% RH and 25 °C. (d) Hydration number at 25 °C over 48 h under various RH conditions. (e) Biological oxygen demand (BOD_t_), BOD of the blank inoculum control (B*_t_*), and biodegradability (BD) of BMDES2 measured over 28 days. (f) Corresponding BOD*_t_*, B*_t_*, and BD profile of BMDES3 under identical test conditions.

Gravimetric analysis quantitatively confirmed this trend. Mass change (Δ*m*) was calculated using eq ([Disp-formula eq1]).

1 $$\Delta m=\frac{m_{t}-m_{0}}{m_{0}}\times 100\left(\%\right)$$

where *m*_0_ denotes the initial mass after drying and *m_t_* represents the mass at time *t*. At 58% RH, pure [Ach]­[Cl] exhibited a mass increase of 51.6% after 48 h, whereas pure [Ph] demonstrated a negligible mass change (Figure 4c). The mass increase progressively decreased with increasing [Ph] content: BMDES1 (20.6%), BMDES2 (12.8%), BMDES3 (9.5%), and BMDES4 (7.6%). The dependence of moisture uptake on RH was further examined at 33% and 75% RH after 48 h (Figure S7a and S7b). At 33% RH, [Ach]­[Cl] exhibited a mass increase of 32.8%, which decreased to 7.0% for BMDES2. Moisture uptake substantially increased at 75% RH, reaching 77.2% for [Ach]­[Cl] and 22.5% for BMDES2, while the compositional trend remained consistent.

To quantify the suppression of moisture uptake, the hydration number (H_2_O/[Ach]­[Cl]) was calculated over 48 h under various RH conditions at 25 °C (Figure 4d). The hydration numbers of pure [Ach]­[Cl] were 3.02, 4.51, and 7.78 at 33, 58, and 75% RH, respectively, indicating strong hygroscopicity. The hydration number systematically decreased with increasing [Ph] content. For example, at 33% RH, the hydration number of [Ach]­[Cl] was 3.02, which decreased to 1.84 for BMDES2, representing a reduction of approximately 39%. A similar trend was observed at higher RH levels, although differences between compositions became less pronounced at 75% RH, where water uptake was dominated by the ambient moisture conditions. Results confirmed that the hydrophobic HBD environment reduced water coordination at ionic sites.

Biodegradability (BD), which was evaluated using the OECD 301C modified Ministry of International Trade and Industry of Japan test (I), was calculated as the ratio of biological oxygen demand at time *t* (BOD*_t_*) to the theoretical oxygen demand (ThOD) after subtraction of the blank control eq ([Disp-formula eq2]).

2 $$\mathrm{BD}=\frac{{\mathrm{BOD}}_{t}-B_{t}}{\mathrm{ThOD}}\times 100\left(\%\right)$$

where B*_t_* is the BOD of the blank inoculum control at time *t*. As shown in Figure 4e,f, the BOD values rapidly increased within the first 5 days and then gradually plateaued over the remainder of the 28-day test period. On day 28, the BOD values reached 287 and 282 mg L^–1^ for BMDES2 and BMDES3, respectively, with corresponding BD values of 97.9 and 92.5%, which exceeded the OECD criterion for ready biodegradability (>60% within 28 days). Results demonstrated that the BMDES samples would readily biodegrade despite their enhanced moisture stability, highlighting the successful integration of environmental robustness with biodegradation performance.

### 2.4 Electrochemical Characteristics of BMDES Systems

Electrochemical impedance spectroscopy (EIS) was performed to evaluate the ionic behavior of BMDES2 and BMDES3 (Figure 5a). BMDES1 and BMDES4 were excluded owing to immiscibility and crystallization issues, respectively, which precluded reliable electrochemical measurement. BMDES2 and BMDES3 exhibited impedance responses that were characteristic of ionic conductors, with low |*Z*| values and large phase angles (*θ*) at high frequencies, transitioning to increased |*Z*| values and reduced *θ* values at low frequencies. BMDES3 displayed slightly lower |*Z*| values than BMDES2 at frequencies below 100 Hz. Ionic conductivity (*σ*) was calculated from resistance at 100 kHz eq ([Disp-formula eq3]).

3 $$\sigma =\frac{t}{AR_{100k}}$$

where *t* denotes the sample thickness, *A* represents the electrode contact area, and *R*_100k_ indicates the resistance at 100 kHz.^33^ The resulting conductivities were 197 and 166 μS cm^–1^ for BMDES2 and BMDES3, respectively (Figure 5b). These values indicated that stoichiometric variation slightly modulated ion transport while preserving overall ionic functionality. The impedance magnitude of BMDES2 decreased at frequencies above 1 Hz, while the phase angle decreased within the frequency range of 1 Hz–10 kHz with increasing temperature as shown in Figure S8a. These results are consistent with the increase in ionic conductivity shown in Figure S8b. As demonstrated in Figure 3h, BMDES2 viscosity strongly depends on temperature. Therefore, the increase in ionic conductivity is primarily attributed to the reduction in viscosity, which facilitates ion transport. Additionally, elevated temperatures may partially disrupt hydrogen-bond and ion–molecule interactions, further enhancing ionic mobility and conductivity.

**Figure 5:**
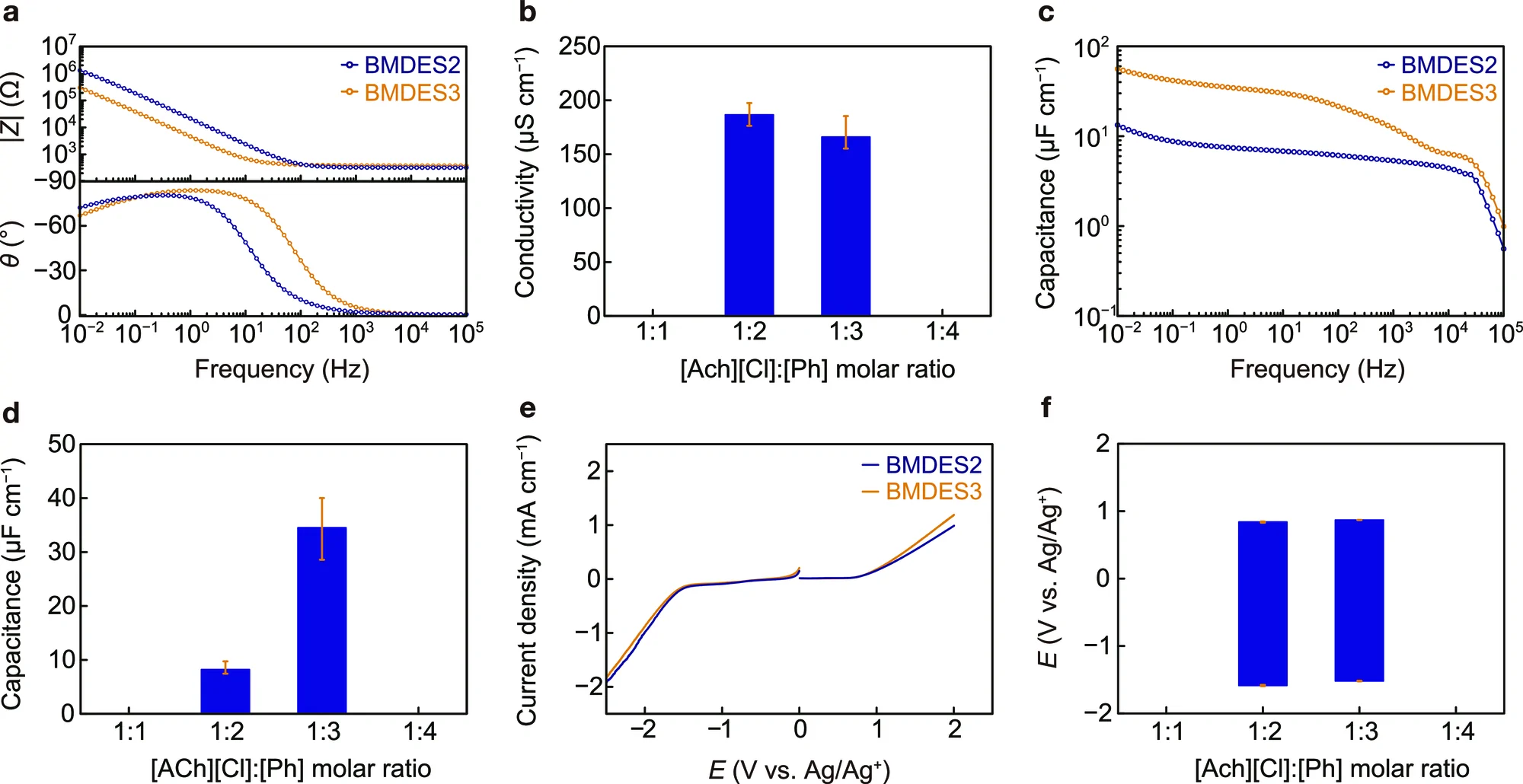
Electrochemical characteristics of stoichiometry-controlled BMBMDES systems. (a) Bode plots of impedance magnitude (|*Z*|) and phase angle (*θ*) for BMDES2 and BMDES3. (b) Ionic conductivity at 100 kHz as a function of HBD content. Error bars represent minimum and maximum values from three independent measurements. (c) Frequency-dependent electrical double-layer capacitance (*C*_EDL_) derived from EIS measurements for BMDES2 and BMDES3. (d) *C*_EDL_ at 1 Hz as a function of HBD content. (e) LSV curves measured in a three-electrode configuration at a scan rate of 5 mV s^–1^ for BMDES2 and BMDES3. (f) Electrochemical stability windows determined from intersection analysis of Faradaic and nonFaradaic regions as a function of HBD content.

Capacitance (*C*) was derived from the imaginary component of impedance eq ([Disp-formula eq4]).

4 $$C=\frac{1}{2\pi f\left|Z_{\mathrm{im}}\right|}$$

The calculated electrical double-layer capacitance (*C*_EDL_) of BMDES3 exceeded that of BMDES2 across the frequency range of 10 mHz to 100 kHz (Figure 5c).^34,35^ At 1 Hz, the *C*_EDL_ values were 9.35 and 34.5 μF cm^–2^ for BMDES2 and BMDES3, respectively (Figure 5d), suggesting enhanced interfacial charge storage with rising [Ph] content.

Linear sweep voltammetry (LSV) was conducted using a three-electrode configuration (glassy carbon working electrode, Pt counter electrode, and Ag/Ag^+^ reference electrode) to assess the electrochemical stability of BMDES2 and BMDES3 (Figure 5e). Potential limits were determined from the intersection of linear fits to the Faradaic and nonFaradaic regions. BMDES2 exhibited anodic and cathodic limits of +0.842 and −1.59 V vs Ag/Ag^+^, respectively, corresponding to a stability window of 2.43 V (Figure 5f). BMDES3 demonstrated a comparable window of 2.39 V vs Ag/Ag^+^. Results confirmed that stoichiometry-induced network reorganization and phase stabilization were compatible with robust ionic conductivity and electrochemical stability.

## 3 Conclusion

This study demonstrates that precise stoichiometric balance governs discrete phase selection in choline-based BMDESs. The hydrophobic HBD acted as a molecular seal, shielding the hygroscopic HBA from moisture through hydrogen-bond network reconfiguration. A glass-forming liquid state was exclusively stabilized at a [Ach]­[Cl] to [Ph] ratio of 1:2, whereas 1:1 or 1:3 ratios resulted in immiscible or crystallizable regimes. Spectroscopic and structural analyses revealed that this behavior was consistent with reorganization of the hydrogen-bond network and altered ion–molecule interactions, leading to short-range ordering without long-range periodicity. Gravimetric analysis and hydration number evaluation indicated that incorporation of the hydrophobic HBD suppressed the mass increase associated with moisture uptake and reduced the number of water molecules coordinated per ionic unit, supporting that stoichiometric variation systematically modulated moisture uptake. Although moisture uptake increased with rising RH, the compositional dependence remained consistent across the tested conditions. Meanwhile, biodegradability and electrochemical functionality were maintained despite enhanced moisture stability. By establishing stoichiometry as a governing parameter for discrete phase selection and moisture regulation—beyond conventional melting point depression—this study provides a rational framework for designing environmentally robust DESs with controlled hydration behavior. This design principle may be extended to other bioderived ionic systems, enabling the systematic tuning of phase stability and environmental compatibility for sustainable electrochemical technologies. Future efforts will focus on extending these bioderived DESs toward flexible sensing and sustainable iontronic applications.

## 4 Experimental Section

### 4.1 Preparation of BMDES Formulations

Acetylcholine chloride ( purity >98.0%, product ID: A0084, Tokyo Chemical Industry Co., Ltd.) and 3-phenylpropionic acid (purity >99%, product ID: W288918, Merck) were purchased and used as received. [Ach]­[Cl] was dried under vacuum (60 °C, 10 Pa) for 24 h prior to use to remove residual moisture. The materials were added to glass vials in a molar ratio of [Ach]­[Cl]:[Ph] = 1:1, 1:1.5, 1:2, 1:3, or 1:4 and heated at 80 °C with magnetic stirring until both components were fully liquefied. After complete liquefaction, the glass vials were kept open and heated at 60 °C under vacuum (10 Pa) for 24 h to remove residual moisture.

### 4.2 FTIR Spectroscopy

FTIR spectra were recorded using a JASCO FT/IR-6XFVST spectrometer equipped with an attenuated total reflection (ATR) accessory and with a diamond ATR crystal. Measurements were conducted under reduced pressure (<1.5 × 10^2^ Pa) to minimize interference from atmospheric moisture and CO_2_. Spectra were collected over the range of 400–4000 cm^–1^. Each spectrum was averaged over 30 scans. Background spectra were recorded prior to each measurement. ATR correction was applied to account for wavelength-dependent penetration depth, and all spectra were baseline-corrected before analysis.

### 4.3 Rheological Measurements

Rheological measurements were performed on BMDES2 and BMDES3 using a rheometer (Anton Paar MCR702-Tg) under a nitrogen atmosphere. The temperature dependence of viscosity for BMDES2 and BMDES3 was recorded at an angular velocity of 1 rad s^–1^, while increasing temperature from 25 to 80 °C at a scan rate of 5 °C min^–1^. The dependence on angular velocity was measured by varying the angular velocity from 0.1 to 100 rad s^–1^ at 25 °C.

### 4.4 X-ray Diffraction

XRD patterns were recorded using a Rigaku SmartLab diffractometer operated in *θ*–2*θ* geometry with Cu K*α* radiation (45 kV, 200 mA). Data were collected over a 2*θ* range of 5–60° with a step size of 0.01° in continuous scan mode at an effective scan rate of 10° min^–1^. Measurements were performed at room temperature.

### 4.5 Moisture Uptake Measurements

Moisture uptake of the individual components and BMDES samples was evaluated under controlled relative humidity conditions at 25 °C. Prior to testing, all samples were dried under vacuum (60 °C, 10 Pa) for 24 h to remove residual moisture. Approximately 500 mg of each sample was placed in open glass Petri dishes (inner diameter: 28 mm) to ensure consistent surface exposure. The dishes were then transferred to sealed containers and stored in an oven maintained at 25 °C with fixed RH levels of 33, 58, or 75%. Relative humidity was established using saturated salt solutions (MgCl_2_·6H_2_O for 33% RH, NaBr for 58% RH, and NaCl for 75% RH) placed at the bottom of the containers. The reported RH values correspond to literature equilibrium values at 25 °C.^36,37^ At predetermined time intervals (1, 2, 6, 24, and 48 h), samples were briefly removed from the containers and weighed using an analytical balance (±0.01 mg, SHIMADZU AP125WD), then returned quickly to minimize disturbance of the humidity environment. Blank controls (empty glass Petri dishes) were measured under identical conditions to correct for possible environmental fluctuations.

### 4.6 Electrochemical Characterization

Chromium (Cr) and gold (Au) with thicknesses of 20 and 200 nm, respectively, were deposited onto a glass substrate via sputtering to create electrodes. Electrode patterns were defined using a stencil mask fabricated by laser cutting. A glass spacer with a 500-μm thickness was used to assemble a pair of electrodes, and the BMDES samples were injected through inlet holes in the spacer. EIS measurements were conducted using an EIS module (Autolab, FRA32M). The temperature of BMDES2 was controlled using a Peltier module equipped with two Pt100 sensors, one for temperature monitoring and the other for feedback control.^24^ Residual oxygen in the BMDESs was purged by bubbling with N_2_ gas for 30 min before performing LSV. LSV for the potential window measurement was performed using a three-electrode cell, with a glassy carbon (EC FRONTIER CO., LTD, catalog ID: GC-3155), platinum (Pt), silver (Ag/Ag^+^, EC FRONTIER CO., LTD, catalog ID: RE-12A), wire electrode (EC FRONTIER CO., LTD, catalog ID: CE-300) as the working, counter, and reference electrodes, respectively. The reference electrode was filled with an acetonitrile electrolyte comprising 10 mM silver nitrate and 100 mM tetrabutylammonium perchlorate. LSV was conducted using a potentiostat (Autolab, PGSTAT204).

## Supplementary Material



## Notes

Photographs of BMDES1.5 before and after cooling (Figure S1); physical properties of BMDES2, including low-temperature stability and nonflammability (Figure S2); photograph of BMDES4 crystallization upon contact with PTFE nuts (Figure S3); phase behavior of DES samples upon contact with DIW (Figure S4); rheological characteristics of BMDES2 and BMDES3 (Figure S5); stoichiometry-dependent moisture stability at 33 and 75% RH (Figure S6); Gravimetric moisture uptake at 33 and 75% RH (Figure S7); temperature-dependent electrochemical properties of BMDES2 (Figure S8) (PDF)
