# Supplementary material for: Stoichiometry-Defined Phase Selection and Hydrophobic Molecular Sealing in Bioderived Deep Eutectic Solvents
Source: ACS Mater Au. 2026 Jun 25;6(5):1141–9. doi: 10.1021/acsmaterialsau.6c00088 (PMC13564151; doi:10.1021/acsmaterialsau.6c00088)
Supplement: Supplementary file 1 [file mg6c00088_si_001.pdf]

Supporting Information

Stoichiometry-Defined Phase Selection and Hydrophobic Molecular Sealing in Bioderived Deep Eutectic Solvents

Shunsuke Yamada[*,†,‡] and Takashi Honda[†]

[†] Department of Electrical and Electronic Engineering, Kyushu Institute of Technology, 1-1 Sensuicho, Tobata, Kitakyushu, Fukuoka 804-8550, Japan

[‡] Center for Advanced Organic-Inorganic Hybrid Materials, Kyushu Institute of Technology, 1-1 Sensuicho, Tobata, Kitakyushu, Fukuoka 804-8550, Japan

Corresponding Author
[*]Shunsuke Yamada
ORCID: 0000-0002-9084-2070
Email: yamada@ele.kyutech.ac.jp

a b

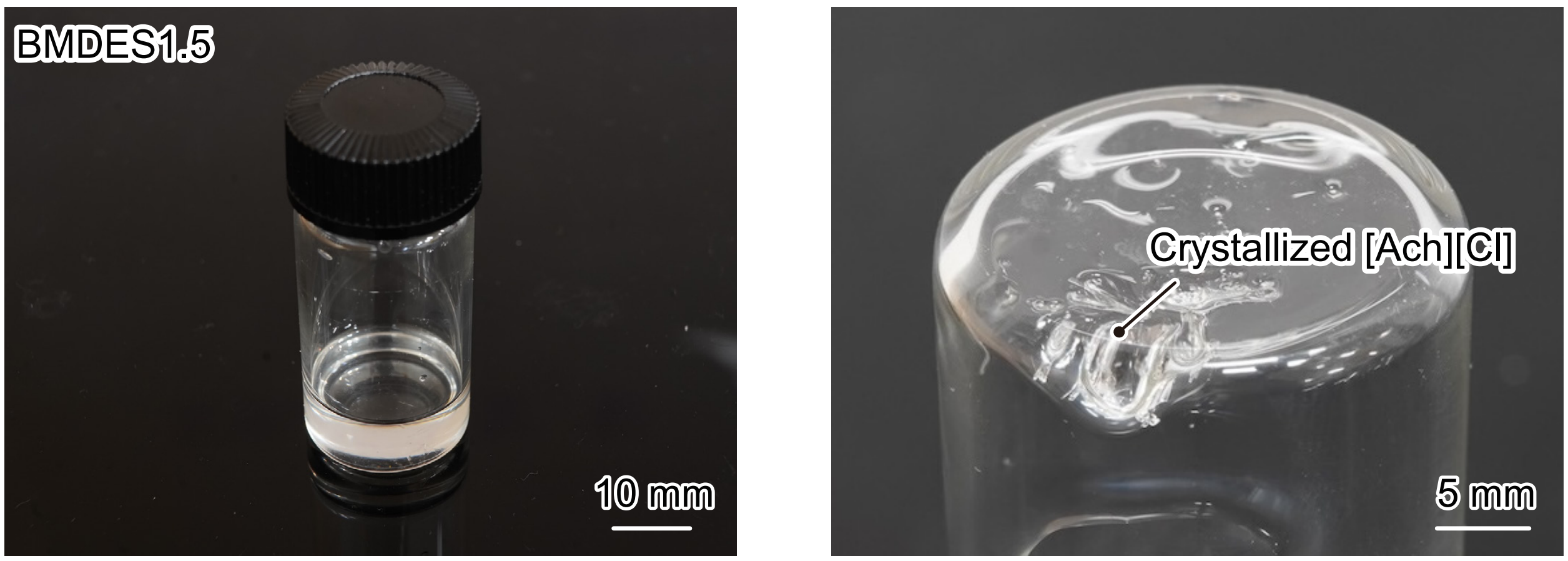

Figure S1. Photographs of BMDES1.5. (a) Appearance after mixing [Ach][Cl] and [Ph] at a 1:1.5 molar ratio and annealing at 80°C. (b) A small amount of crystallized [Ach][Cl] was observed after cooling to room temperature.

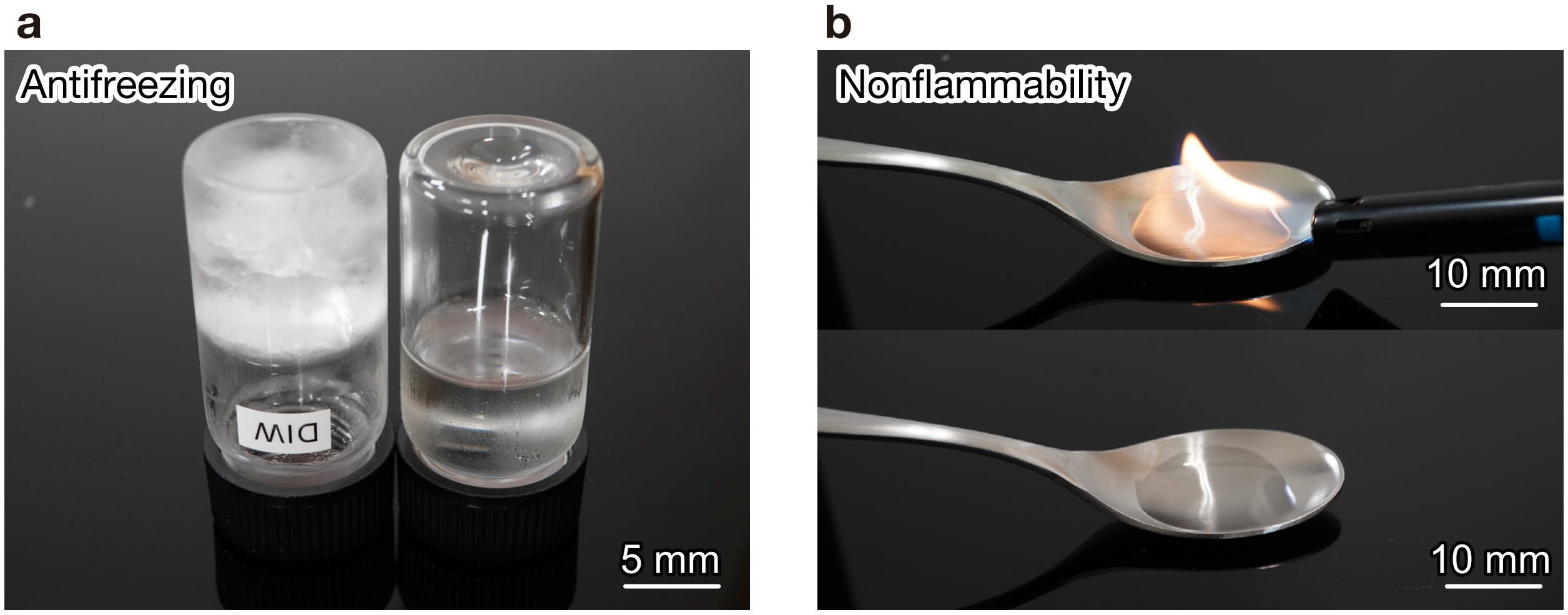

Figure S2. Physical properties of BMDES2. (a) Liquid state retained at −20°C, confirming liquid-state retention in contrast to solidified deionized water used as reference. (b) Non-flammability upon direct flame exposure.

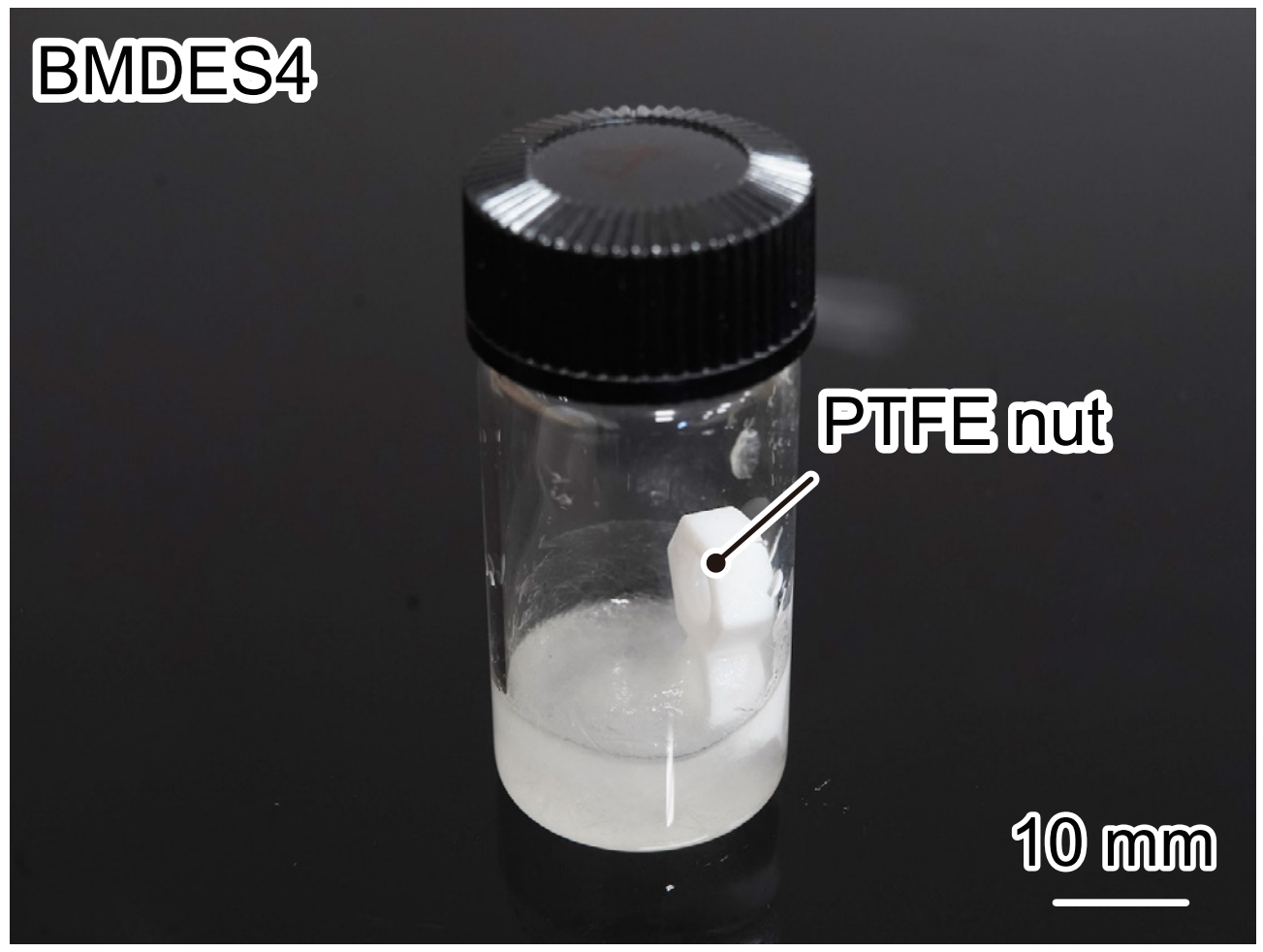

Figure S3. Photograph of BMDES4. BMDES4 crystallized upon contact with PTFE nuts.

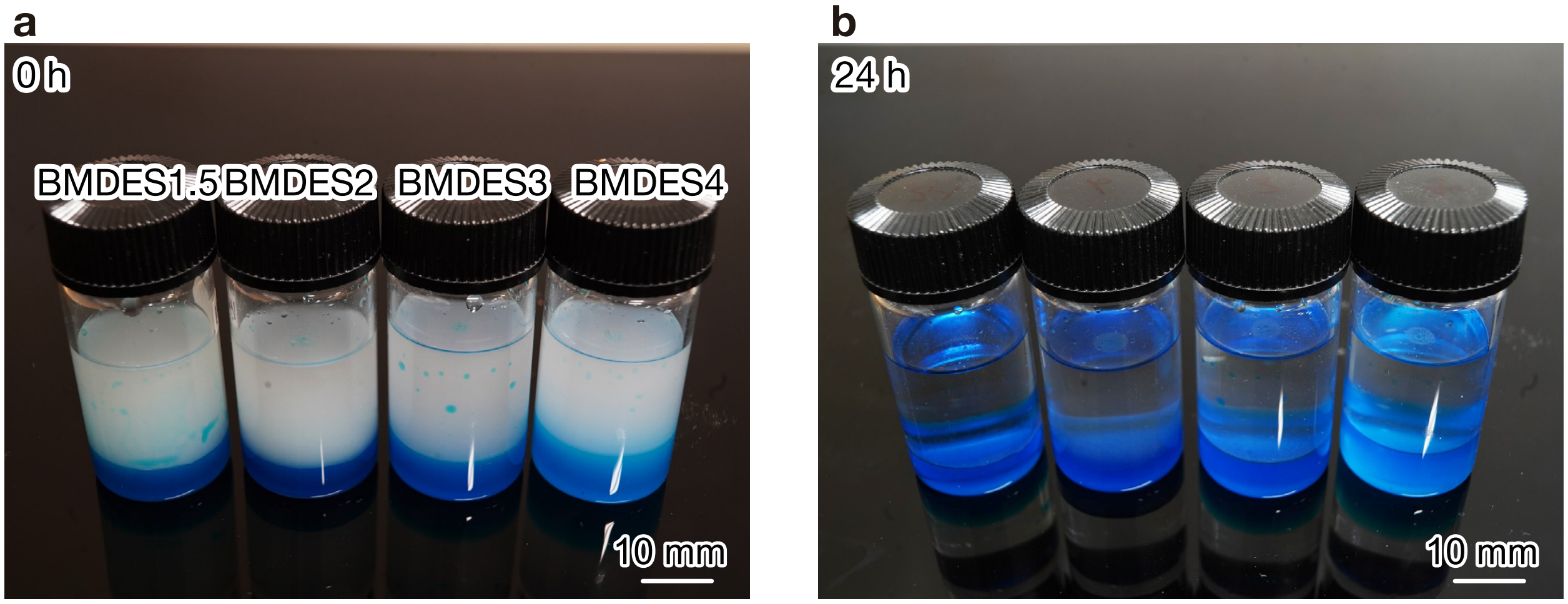

Figure S4. Phase behavior upon water contact. (a) BMDES1.5, BMDES2, BMDES3, and BMDES4 formed biphasic systems with an emulsion-containing aqueous layer and a BMDES-rich layer. (b) Time-lapse showing emulsion settling and coalescence into a clear aqueous phase. The BMDESs were dyed blue for visual clarity.

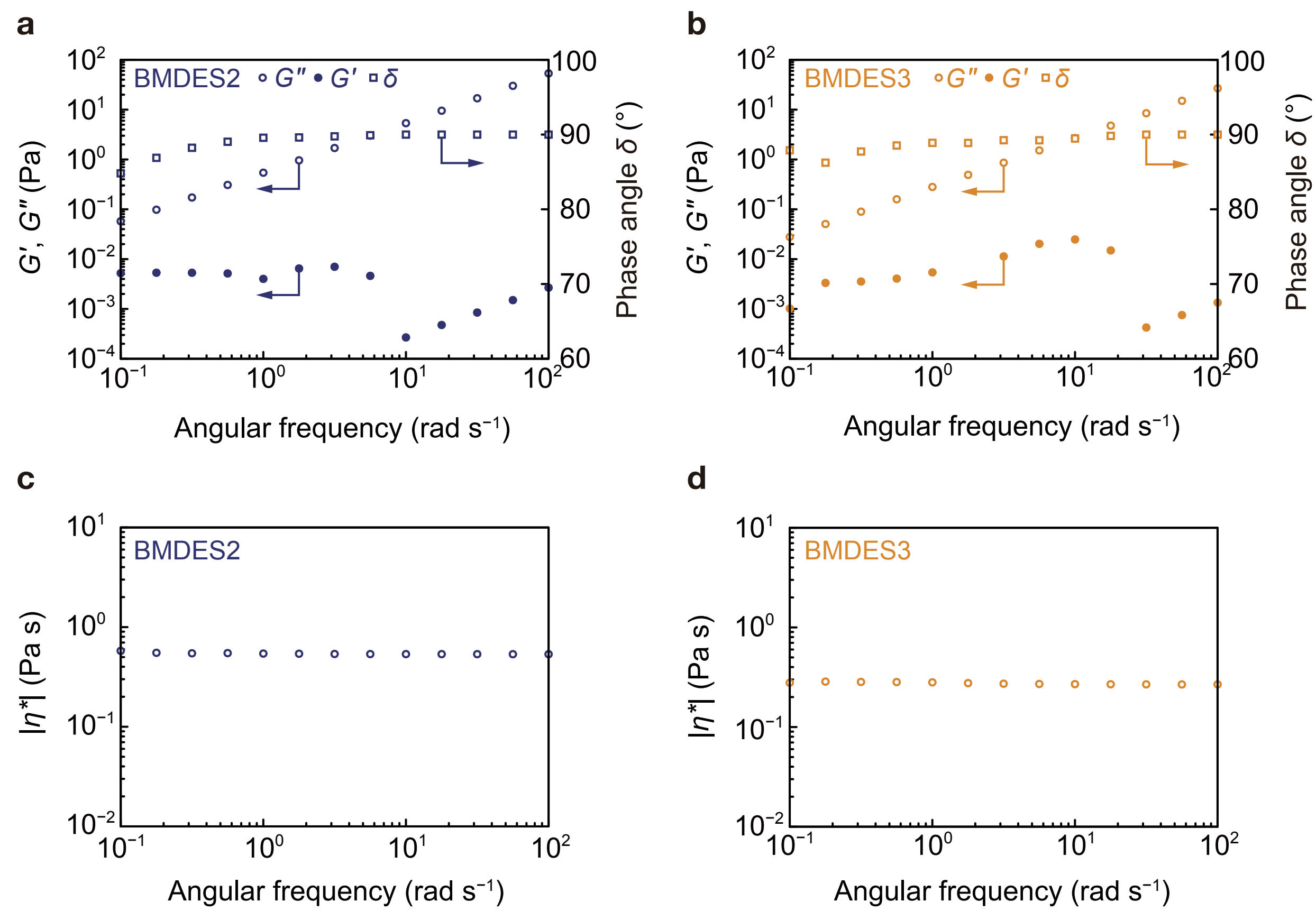

Figure S5. Rheological characteristics of the BMDESs. Storage modulus (G'), loss modulus (G''), and phase angle (δ) of BMDES2 (a) and BMDES3 (b), and complex viscosity (η*) of BMDES2 (c) and BMDES3 (d), as functions of angular frequency.

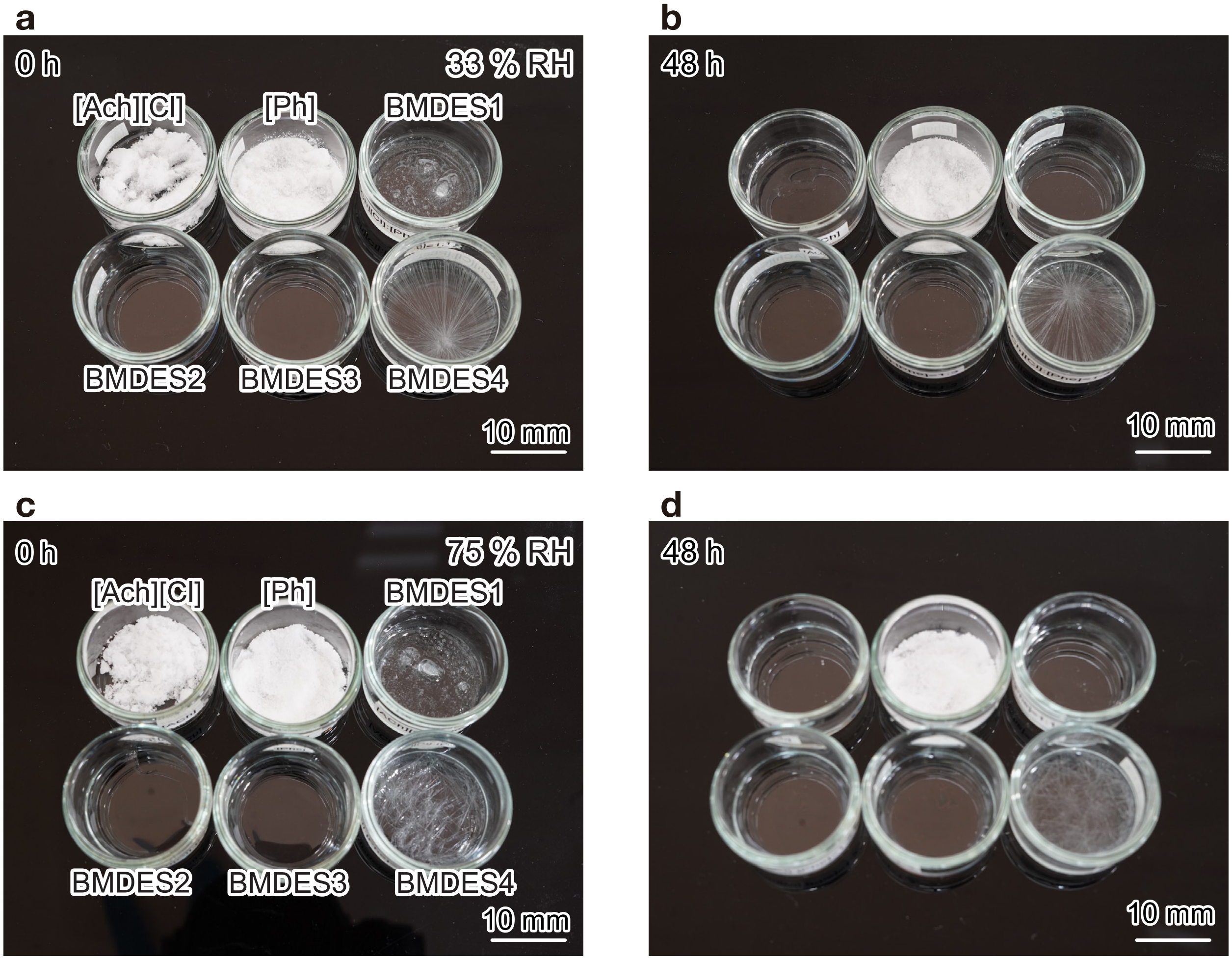

Figure S6. Stoichiometry-dependent moisture stability. Photographs of [Ach][Cl], [Ph], BMDES1, BMDES2, BMDES3, and BMDES4 before (a, c) and after (b, d) 48 h exposure at 25°C under 33% RH (a, b) and 75% RH (c, d). [Ach][Cl] undergoes deliquescence and BMDES1 shows partial dissolution, whereas BMDES2, BMDES3, and [Ph] maintain structural integrity. BMDES4 exhibits crystallization upon transfer.

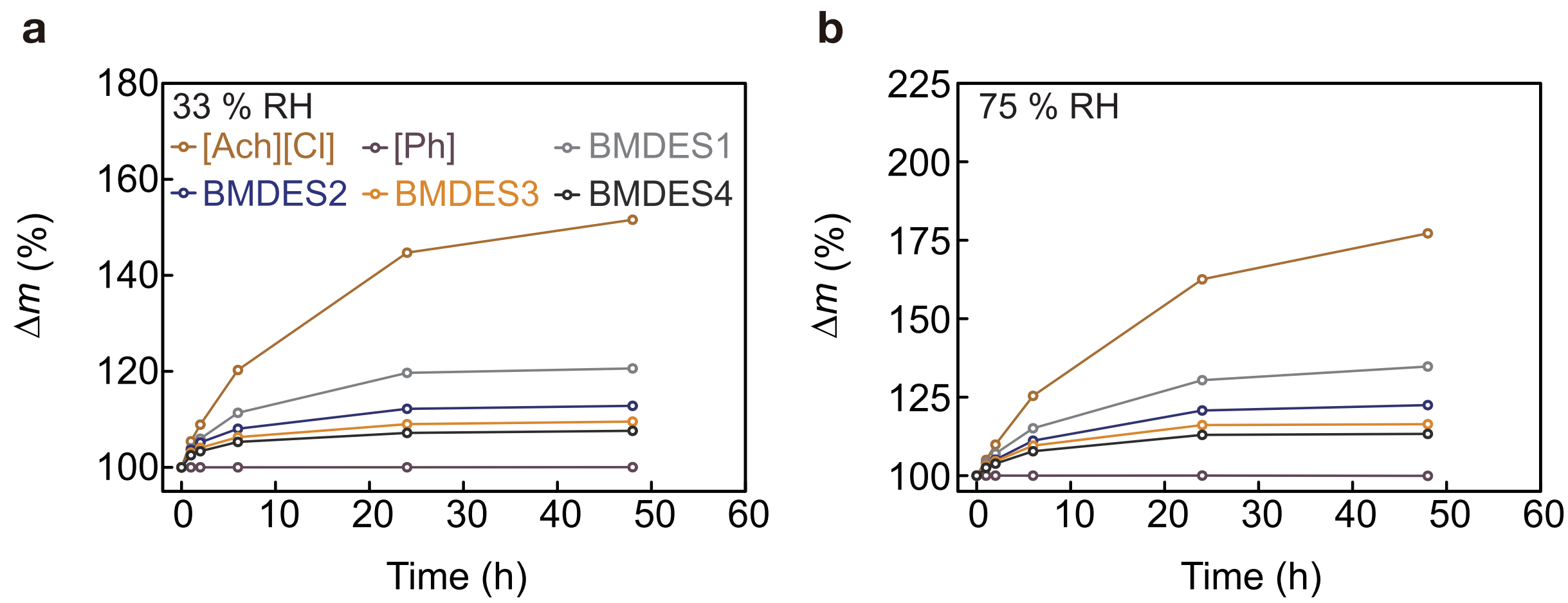

Figure S7. Humidity-dependent moisture uptake of BMDES compositions at 25°C over 48 h.

(a) 33% RH. (b) 75% RH. Mass change (Δm) was calculated according to equation (1)

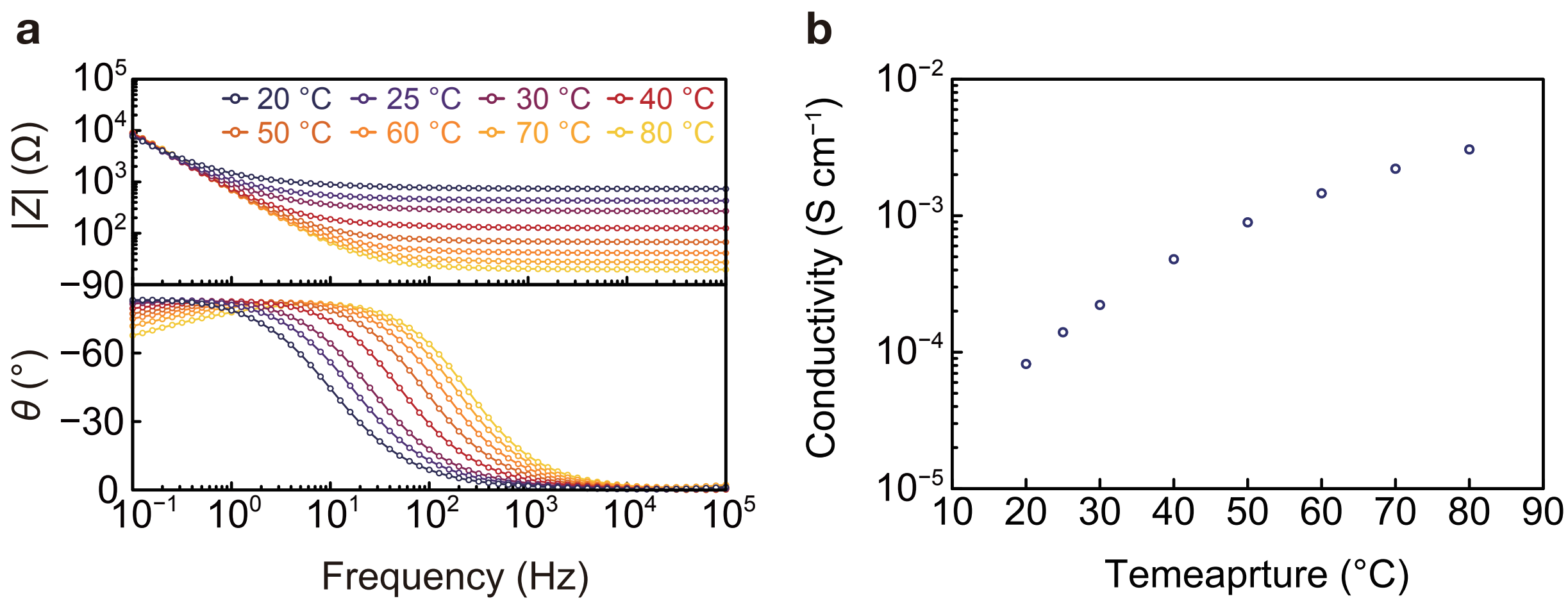

Figure S8. Temperature-dependent electrochemical properties of BMDES2. EIS spectra recorded at various temperatures (a) and the corresponding ionic conductivity values determined at 100 kHz (b).
